# Supplementary material for: Light-Trapping Electrode for the Efficiency Enhancement of Bifacial Perovskite Solar Cells
Source: Nanomaterials (Basel). 2022 Sep 15;12(18):3210. doi: 10.3390/nano12183210 (PMC9500818; doi:10.3390/nano12183210)
Supplement: Supplementary file 1 [file nanomaterials-12-03210-s001.zip › nanomaterials-1876463-suplemmentary.pdf]

Supplementary Materials: Light-Trapping Electrode for the Efficiency Enhancement of Bifacial Perovskite Solar Cells

Anna A. Obraztsova [1,*,†], Daniele Barettin [2,*,†], Aleksandra D. Furasova [1], Pavel M. Voroshilov [1], Matthias Auf der Maur [3], Andrea Orsini [2] and Sergey V. Makarov [1,4,5]

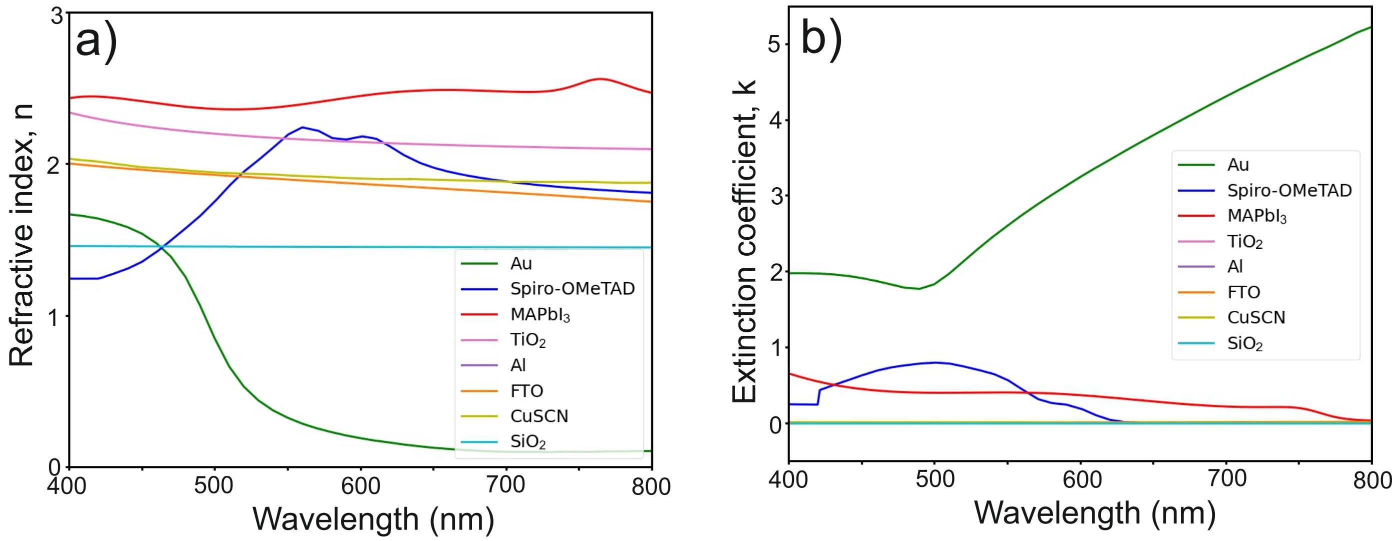

Figure S1. The complex refractive index of each materials (**a**) refractive indexes, (**b**) extinction coefficients.

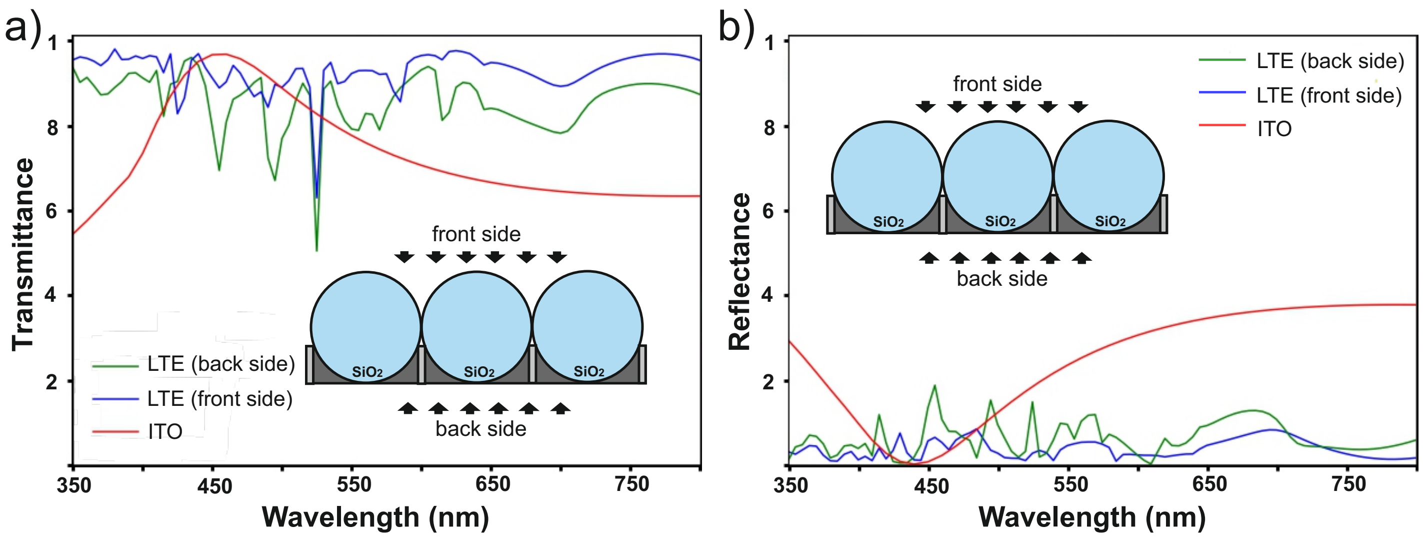

Figure S2. Optical characteristics for light trapping electrode and ITO in comparison: (**a**) transmittance, (**b**) reflectance.

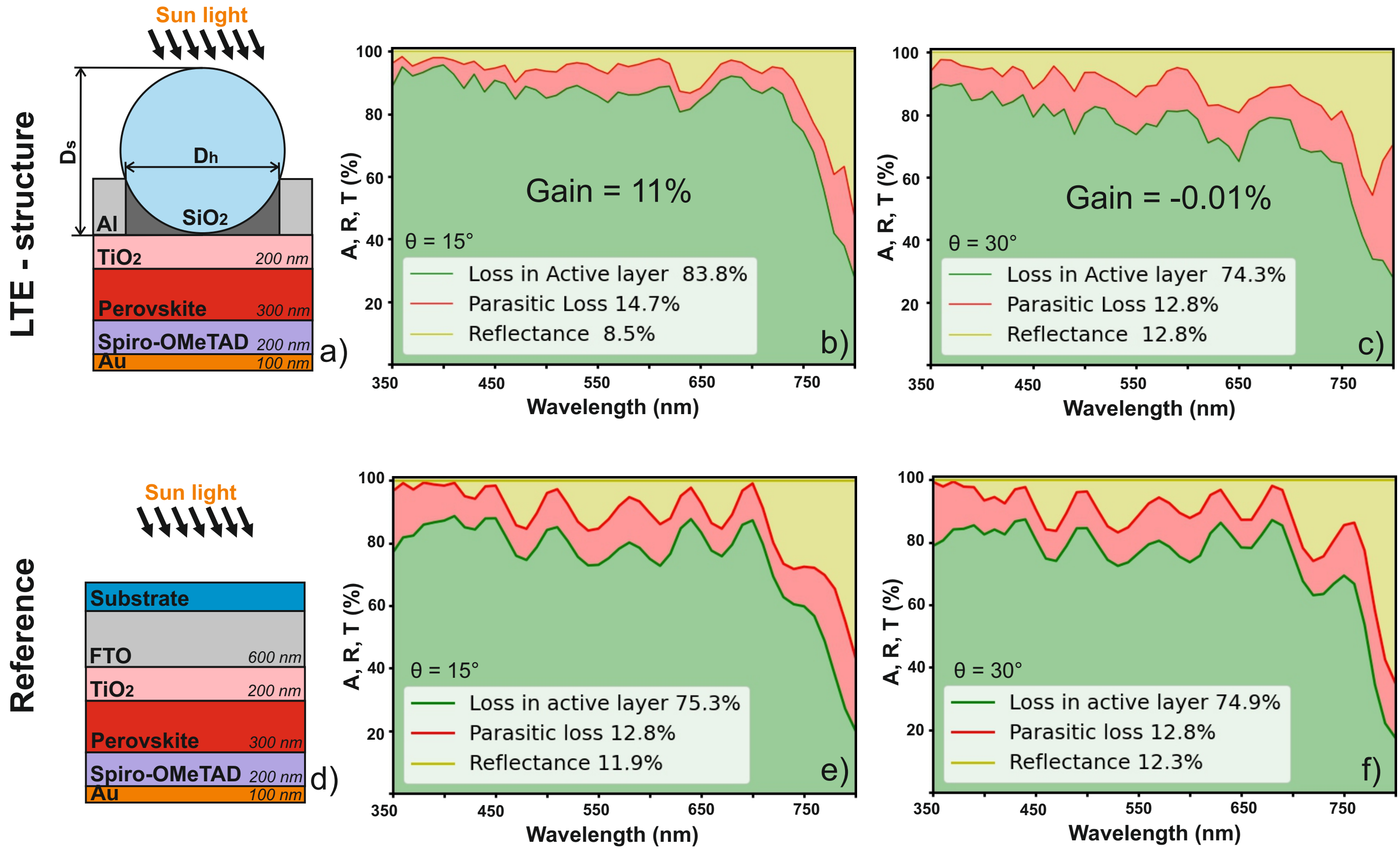

Figure S3. Optical efficiency analysis of monofacial perovskite solar cells under oblique light incidence (calculated for $\lambda = 350 - 800$ nm). (**a**) The scheme of light trapping structure for the monofacial cell and its (**b**) spectral absorption, transmission, and reflection parameters obtained under an angle of incidence $\Theta = 15°$, (**c**) calculated spectral absorption, transmission, and reflection of light trapping structure for the monofacial perovskite solar cell under angle of incidence $\Theta = 30°$(**d**) the scheme of the considered reference monofacial cell, (**e**) spectral absorption, transmission, and reflection of the reference monofacial cell structure under angle of incidence $\Theta = 15°$ and under $\Theta = 30°$ (**f**).

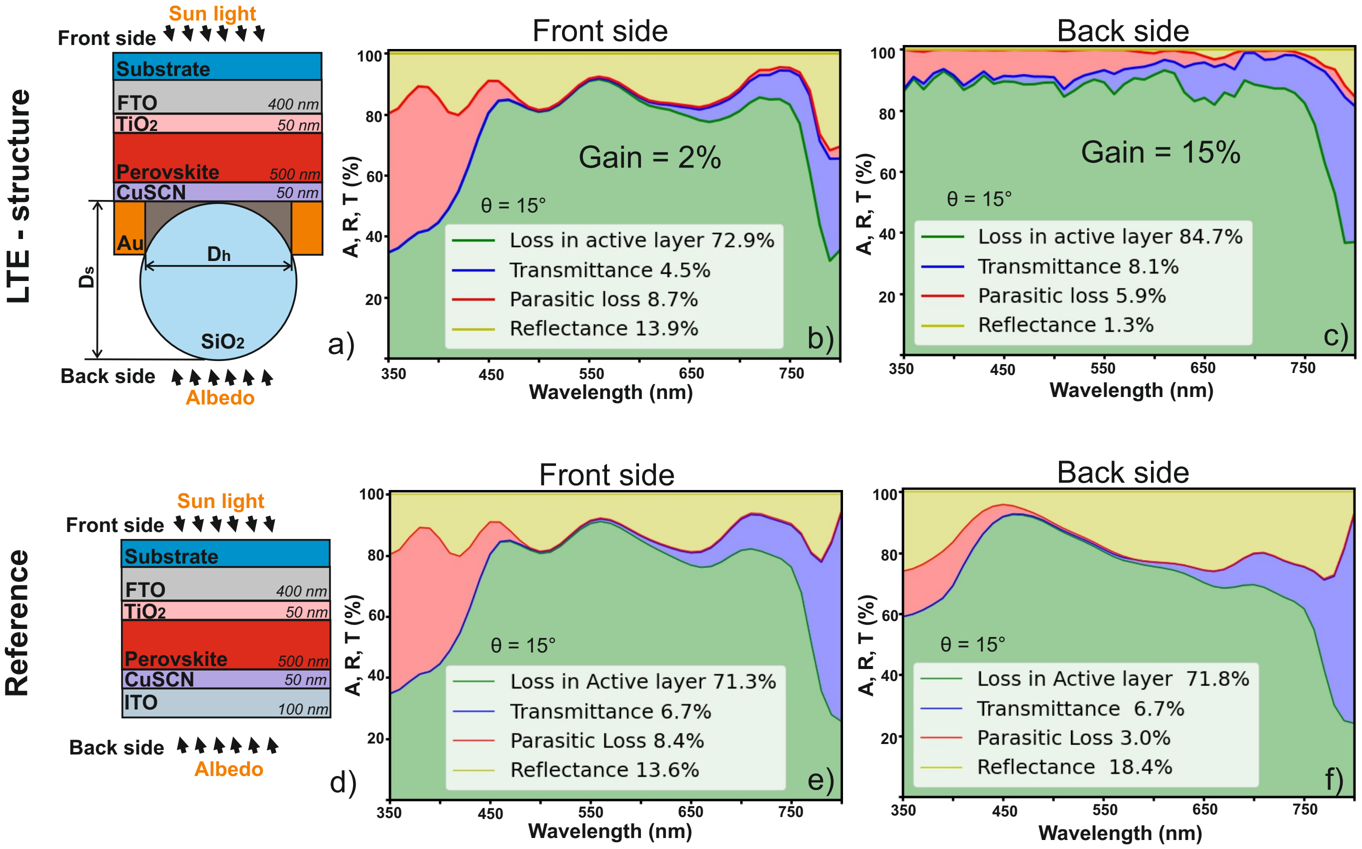

Figure S4. Optical efficiency analysis of the bifacial perovskite solar cell under light illumination with spectral range of $\lambda = 350 - 800$ nm with an angle of incidence $\Theta = 15°$. (**a**) The scheme of the considered light trapping structure for bifacial perovskite solar cell, (**b**) calculated spectral absorption, transmission, and reflection for the front side of light trapping structure for the bifacial cell, (**c**) spectral absorption, transmission, and reflection reached from the back side of light trapping structure of the bifacial cell, (**d**) the structure of the reference bifacial perovskite solar cell, (**e**) spectral absorption, transmission, and reflection for the front side of reference and optical parameters reached from the back side (**f**).

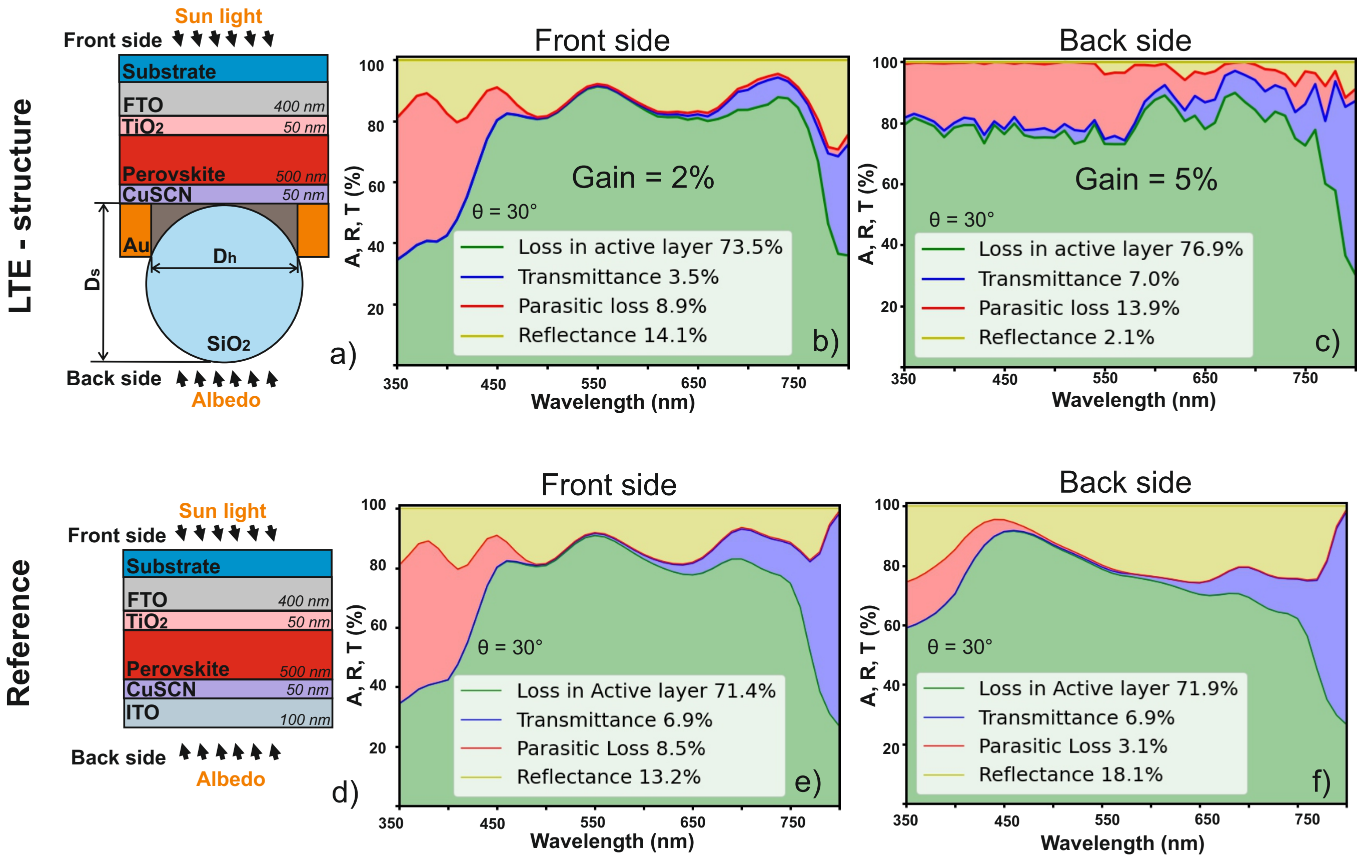

Figure S5. Calculated optical efficiency analysis of bifacial PSCs exposed to light illumination with $\lambda = 350 - 800nm$ with an angle of incidence $\Theta = 30°$. (**a**) The scheme of the light trapping structure for the bifacial perovskite solar cell, (**b**) spectral absorption, transmission, and reflection for the front side of light trapping structure for the bifacial device, (**c**) spectral absorption, transmission, and reflection for the back side of the light trapping structure for the bifacial cell, (**d**) the scheme of the reference bifacial cell and its (**e**) spectral absorption, transmission, and reflection for the front side and (**f**) spectral absorption, transmission, and reflection under illumination from the back side.
